# Core transcriptional signatures of phase change in the migratory locust

**DOI:** 10.1007/s13238-019-0648-6

**Published:** 2019-07-10

**Authors:** Pengcheng Yang, Li Hou, Xianhui Wang, Le Kang

**Affiliations:** 1 Beijing Institutes of Life Science, Chinese Academy of Sciences, Beijing, 100101 China; 2 State Key Laboratory of Integrated Management of Pest Insects and Rodents, Institute of Zoology, Chinese Academy of Sciences, Beijing, 100101 China

**Keywords:** phenotypic plasticity, transcriptional regulatory network, RNA interference

## Abstract

Phenotypic plasticity plays fundamental roles in successful adaptation of animals in response to environmental variations. Here, to reveal the transcriptome reprogramming in locust phase change, a typical phenotypic plasticity, we conducted a comprehensive analysis of multiple phase-related transcriptomic datasets of the migratory locust. We defined PhaseCore genes according to their contribution to phase differentiation by the adjustment for confounding principal components analysis algorithm (AC-PCA). Compared with other genes, PhaseCore genes predicted phase status with over 87.5% accuracy and displayed more unique gene attributes including the faster evolution rate, higher CpG content and higher specific expression level. Then, we identified 20 transcription factors (TFs) named PhaseCoreTF genes that are associated with the regulation of PhaseCore genes. Finally, we experimentally verified the regulatory roles of three representative TFs (*Hr4*, *Hr46*, and *grh*) in phase change by RNAi. Our findings revealed that core transcriptional signatures are involved in the global regulation of locust phase changes, suggesting a potential common mechanism underlying phenotypic plasticity in insects. The expression and network data are accessible in an online resource called LocustMine (http://www.locustmine.org:8080/locustmine).

**Electronic supplementary material:**

The online version of this article (10.1007/s13238-019-0648-6) contains supplementary material, which is available to authorized users.

## INTRODUCTION

Phenotypic plasticity is prevalent in organisms and enables individuals of the same species to develop alternative phenotypes in response to changing environments with same genotype (West-Eberhard, 2003). Phenotypic plasticity is usually characterized by remarkable changes in various biological traits, including morphological, behavioral traits, and so on (Pigliucci, 2001; DeWitt and Scheiner, 2004). Moreover, these changes often affect entire suites of characters in numerous tissues throughout life (Pigliucci, 2001; DeWitt and Scheiner, 2004). To reveal the transcriptional regulatory mechanisms of such a complex natural phenomenon, a number of studies have identified numerous differentially expressed genes (DEGs) related to phenotypic plasticity in some of model species utilizing high-throughput gene expression profiling technologies (Zayed and Robinson, 2012; Dal Santo et al., 2013; Le Trionnaire et al., 2013; Brown et al., 2014). Comparative transcriptome analysis further revealed that a small core set of genes are consistently associated with specific phenotypes across various tissues (Johnson and Jasper, 2016) and developmental stages (Morandin et al., 2015) within species, and some gene modules regulating similar behavior are conserved among species (Corona et al., 2016). In addition, transcriptional regulatory network (TRN) analysis found core transcription factors (TFs) could globally regulate behavior difference (Chandrasekaran et al., 2011). Using different study systems, many researchers have reported that these phenotypic plasticity-related genes display unique characteristics associated with several investigated features, such as faster evolution rate (Hunt et al., 2011), higher CpG content (Elango et al., 2009), and lower DNA methylation level (Patalano et al., 2015). However, few studies have explored the gene features and regulatory roles of the core transcriptional signatures in one organism across spatiotemporal scales (Schlichting and Smith, 2002).

The migratory locust, *Locusta migratoria*, displays a remarkable density-dependent phase change, a typical phenotypic plasticity, involving the transition between solitary and gregarious phases (Pener and Simpson, 2009; Wang and Kang, 2014). Locust individuals can shift multiple phase-related traits, such as body color, behavior, metabolic and hormonal physiology, immune function, and reproduction in response to the changes of population density (Pener and Simpson, 2009). By a variety of comparative omics analyses, several key regulatory genes and small RNAs, have been revealed to be involved in the regulation of such complex phase-related traits, including body color (Yang et al., 2019), behavior (Guo et al., 2011; Ma et al., 2011; Wu et al., 2012; Hou et al., 2017), immunity (Wang et al., 2013), or reproduction (He et al., 2016). In particularly, we have accumulated numerous high-throughput transcriptome datasets from various tissues, developmental stages, and time courses of phase transition (Chen et al., 2010; Wang et al., 2013; Wang et al., 2014; Chen et al., 2015; Yang et al., 2019). And, the sequenced locust genome further provided more genomic information and reference sequences (Wang et al., 2014). Therefore, the migratory locust is used as an ideal model to investigate the core transcriptional signatures involved in the regulation of phenotypic plasticity across various spatiotemporal scales through integrative transcriptome analysis.

Integrative transcriptome analysis is one kind of horizontal genomic meta-analysis combining one source of -omics information (Tseng et al., 2012; Kapheim, 2016). Many methods have been developed to tackle the issues encountered during integrative transcriptome analysis, such as confounding factors removing (Lin et al., 2016), ranks aggregation (Li et al., 2019) and TRN construction (Marbach et al., 2012). Integrative transcriptome analysis has been widely applied for the detections of DEGs, pathways, networks or gene co-expression (Rhodes and Chinnaiyan, 2005; Tseng et al., 2012) due to its higher statistical power by increasing sample size (Normand, 1999). By integrating transcriptome datasets from multiple treatments, some candidate genes responsible for behavioral maturation are identified in honey bee (Whitfield et al., 2006) and the core transcriptional responses under numerous environmental and genetic perturbations are determined in *Synechocystis* (Singh et al., 2010). The method constructing tissue-to-tissue co-expression networks can give help for the identification of obesity-specific subnetworks responding to changes in different tissues (Dobrin et al., 2009). Therefore, the development of these analysis tools and successful applications provide a chance to reveal the core transcriptional signatures and their regulatory roles in locust phase change.

In this study, we took the use of adjust confounding principal component analysis (AC-PCA) (Lin et al., 2016) to remove the confounding factors and performed gene selection for multiple locust transcriptomic datasets. Then, we identified PhaseCore genes and PhaseCoreTF genes associated with locust phase change, and verified these genes’ reliability and function through both dry and wet experiments. Our results support there exist core transcriptional signatures across spatiotemporal scales responsible for phenotypic plasticity in one species.

## RESULTS

### Dataset establishment

**Figure 1:**
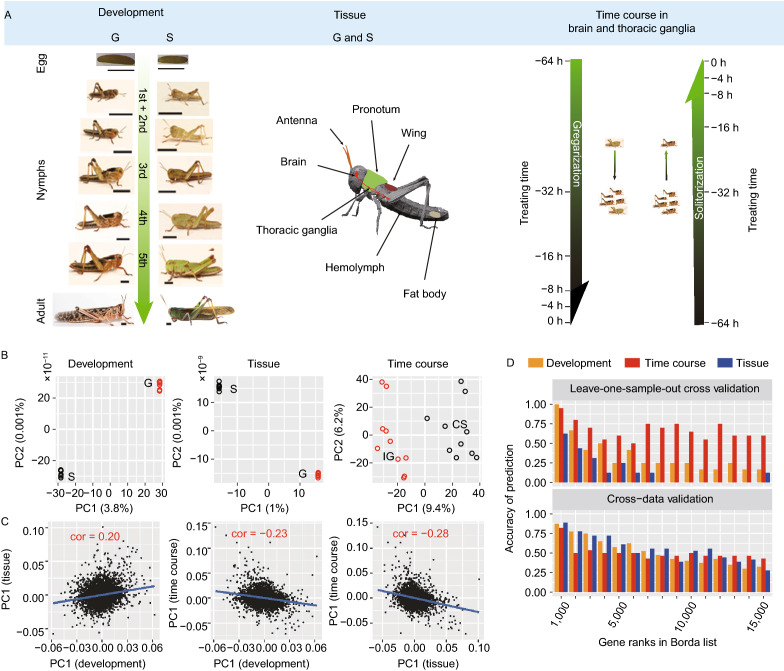
**PhaseCore gene identification**. (A) Experimental design of this study. Left: developmental stages from eggs to adults. Scale bars = 5 mm. Middle: various tissues, including three tissues from adult locust (fat body, hemolymph, and antenna), and five tissues from the fourth instar nymphs (antenna, brain, thoracic ganglia, wing, and pronotum). Right: the time courses of phase change (i.e., gregarization and solitarization) with two brain and thoracic ganglia tissues at six time points (0, 4, 8, 16, 32, and 64 h). (B) Samples from gregarious (G) and solitary locusts (S), and CS and IG locusts classified using the AC-PCA method for developmental, tissue, and time course datasets. One circle represents one sample. Blue represents typical or crowded solitary locusts, and red represents typical or isolated gregarious locusts. (C) Scatterplots and Pearson’s correlation (marked in red) of pairs of the PC1 values from the three datasets. Lines were fitted using least-squares linear regression. (D) Accuracy distribution of leave-one-out cross validation (LOO-CV) and cross-dataset validation (CDV) for the three datasets using Borda gene list. Only the top 15,000 genes were considered. These genes were divided into 15 bins with 1,000 genes in each bin. The accuracy was calculated for each bin

In the past decade, we have accumulated numerous high-throughput transcriptome datasets from various tissues, developmental stages, and time courses of phase transition of the migratory locust. The datasets provided the possibility for us to identify the core genes closely associated with phase changes termed PhaseCore genes. To identify these core genes, we firstly established three transcriptomic datasets (see MATERIALS AND METHODS) (Fig. 1A and Table S1). Three categories of transcriptomic datasets included a developmental dataset from egg to adult stages (Chen et al., 2010), tissue dataset of eight tissues or organs (brain, thoracic ganglia, antennae, wing, pronotum, fat body, and hemolymph), and time course datasets of brain and ganglia tissues treated by gregarization (crowding of solitary locusts (CS)) and solitarization (isolation of gregarious locusts (IG)) (see MATERIALS AND METHODS). These samples covered 97.4% of the 17,586 genes in the reference gene set with reads per kilobase per million reads (RPKM) > 0 in any one sample (Table S2). Hierarchical cluster analysis and principal component analysis (PCA) showed that the transcriptome RNA-seq samples from the same developmental stages or same tissues/organs clearly clustered together (Fig. S1), indicating that the datasets were reliable.

### PhaseCore gene identification

To rank the genes according to their contribution to the phase difference, we then tested whether two popular clustering algorithms, unsupervised PCA and supervised partial least square regression could classify all RNA-seq samples in consistent with their phase status. However, these two methods did not clearly classify the transcriptome samples into two groups, probably because of confounding factors (Figs. S1 and S2). Then, we conducted a recently developed method, AC-PCA, which extends the conventional PCA by adjusting the confounding factor (Lin et al., 2016). The dominant principal component (PC1), which explained larger proportions of variance than PC2 (3.8% vs. <0.001% in the development dataset, 1% vs. <0.001% in the tissue dataset, and 9.4% vs. 6.2% in the time course datasets), clearly classified all the three datasets into gregarious and solitary groups (Fig. 1A). Thus, PC1 represents the difference of gene expression between the phase-related features. Moreover, the PC1 value lists from all three datasets were correlated with each other (Fig. 1B). Therefore, three gene lists from the three datasets were ranked based on PC1 values and aggregated by the Borda algorithm to produce one ranked gene list (the Borda list).

**Figure 2:**
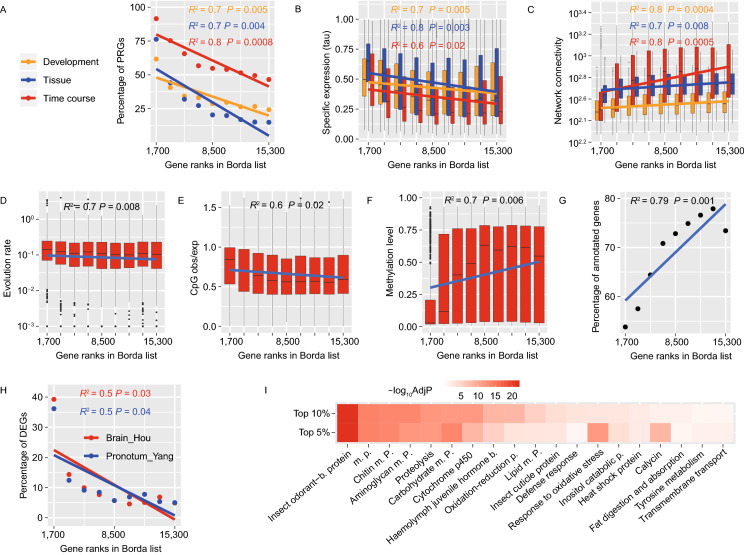
**The attributes and functions of PhaseCore genes**. PhaseCore genes displayed extreme gene attributes (A–H). The PhaseCore gene sets were the top 1,700 genes in the Borda gene list (i.e., the far-left column in (A–H)). PhaseCore genes displayed (A) higher percentage of PRGs, (B) higher specific expression level, (C) lower network connectivity in the co-expression network, (D) faster evolution rate, (E) higher CpG o/e, (F) lower methylation level, (G) lower percentage of genes with known function and (H) higher percentage of DEGs from two experiments with three replicates. (I) Selected enriched functional classes of PhaseCore genes at two cutoff: 10% and 5%. Red represents the degree of the enrichment. b. binding; p. process; m. metabolic; c. compound

To predict how many genes associated with phase-related features across the three investigated datasets, we obtained a cutoff to define the core gene set using two cross validation methods: leave-one-sample-out cross validation (LOO-CV) and cross-dataset validation (CDV), and functional categories enrichment analysis. We first split the top-ranked 15,000 genes from the four ranked gene lists (three dataset-specific gene lists and one Borda list)) into 15, 30, and 150 bins with 1,000, 500, and 100 genes per bin, respectively, and then performed validation for each bin. Through cross validation, we found that the prediction accuracy of the top ranked 2,000 genes was at least ~50% for the Borda list (Figs. 1C, S3 and S4A), Functional categories enrichment analysis of the top 5% and 10% (850 and 1,700 genes, respectively) of the total genes in the genome. We did not find that the top-ranked genes in the top 5% cutoff were more significantly enriched in gene functional categories than those genes in the top 10% cutoff (Fig. 2I). The prediction accuracy of LOO-CV using 1,700 PhaseCore genes was 87.5%. Therefore, we finally defined 1,700 PhaseCore genes using the top 10% as the cutoff in the Borda list (Table S3).

### PhaseCore gene features

To validate the reliability of PhaseCore genes, we performed a multiple comparison test between PhaseCore genes and all other non-PhaseCore genes. The multiple comparison test evaluated seven measurements, including the percentage of phase-related genes (PRGs) (Fig. S5), specific expression, network connectivity, evolution rate, ratio of observation to expectation of CpG (CpG o/e), methylation level, and percentage of genes with known function.

To compare the PhaseCore genes with other non-PhaseCore genes, we selected the top 15,300 genes (because the minimum number of genes in the three datasets was 15,360) in the Borda gene lists. These genes were then sequentially divided into nine bins, with 1,700 genes in each bin, hence the genes in the first bin were the PhaseCore genes. For each bin, we calculated the seven measurements and plotted the measurement distribution along with the rank of the genes. Compared with non-PhaseCore genes, PhaseCore genes showed higher percentage of PRGs (Fig. 2A), higher specific expression level (Fig. 2B), lower co-expression network connectivity (Fig. 2C), faster evolution rate (Fig. 2D), higher CpG o/e (Fig. 2E), lower methylation level (Fig. 2F), and lower percentage of genes with annotated function (Fig. 2G). Similar patterns were also observed in the three dataset-specific gene lists (Fig. S4B–H). These data indicated that PhaseCore genes displayed unique characteristics.

Because these features were also observed in the plasticity-related genes of other species, we speculated that PhaseCore genes are mostly correlated with locust phase change. So we validated PhaseCore genes using the DEGs from two studies with three replicates in brain and pronotum tissues (hence named Brain_Hou and Pronotum_Yang, respectively) (Hou et al., 2017; Yang et al., 2019). We found that PhaseCore genes significantly cover more DEGs than other genes (hypergeometric test, *P* < 1 × 10^−70^ for both studies; Fig. 2H). This pattern was also observed in the three dataset-specific gene lists (Fig. S4I–K).

Functional class enrichments of PhaseCore genes showed significant associations with signalling pathways, metabolic processes, anti-oxidative processes, and structural constituent of cuticle (Fig. 2I and Table S4). We also found that 47.5% of the genes involved in juvenile hormone (JH) biosynthesis and transportation, such as *JHBP*, *JHAMT*, and *HexL1*, were PhaseCore genes (Table S5).

### PhaseCoreTF genes and their regulatory network

**Figure 3:**
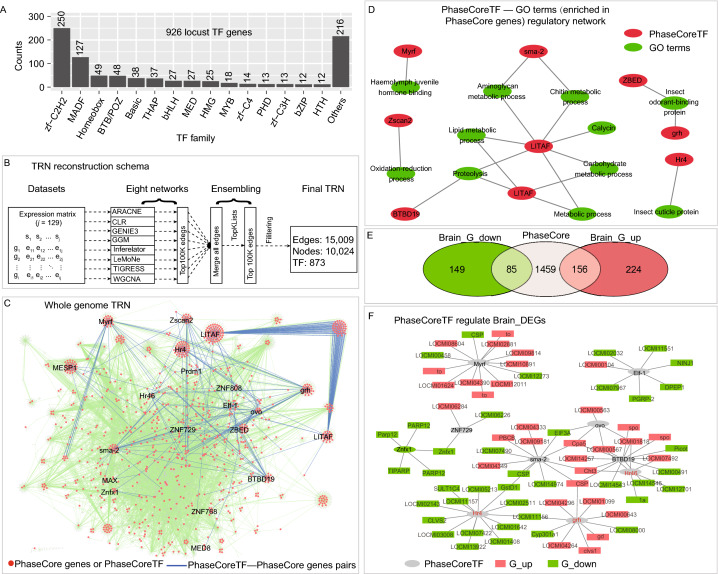
**Identification and regulational functions of PhaseCoreTF genes**. (A) Barplot of locust TF families with >10 members. (B) Schema of transcriptional regulatory network (TRN) reconstruction. (C) Whole genome TRN. The red nodes represent the PhaseCore genes or PhaseCore TF genes, which were connected by green lines. The labelled nodes were 20 PhaseCore TF genes. (D) PhaseCoreTF regulating GO terms enriched in PhaseCore genes. (E) Venn diagram displaying the overlap among the DEGs from Brain_Hou dataset and PhaseCore genes. (F) Network presentation of PhaseCoreTF regulating PhaseCore genes. Ellipse nodes are TF genes, rectangle nodes are target genes. Nodes in red or green represent highly or lowly expressed in gregarious locust, and gray represents non-DEGs

To find the TF gene regulating PhaseCore genes, we conducted a genome-wide investigation of TF genes and constructed a TRN composed of all TF genes and their target genes. Based on the locust genome (Wang et al., 2014), we identified 926 TF genes that could be classified into 94 families, in which zinc finger C2H2, MADF, and homeobox families having the most numbers of members (Fig. 3A). We found that 52.9% (*n* = 490) of TF genes were PRGs (Fig. S6). There were 33 TF genes in the PhaseCore genes. These results implied that TF genes play critical roles in locust phase change.

To construct a reliable and robust genome-wide TRN with the heterogeneous datasets, we combined eight high-performing methods for TRN reconstruction with an ensemble method to integrate these results (Fig. 3B). The heterogeneous datasets were composed of 129 samples, which included 48 samples from three datasets mentioned above and 81 additional samples produced in our laboratory (Table S2). In the final network, 10,024 nodes were connected by 15,009 edges (Fig. 3C). These nodes covered 873 TF genes, and 986 PhaseCore genes. On average, each TF regulated 17.2 target genes.

Based on the genome-wide TRN, we defined PhaseCoreTF genes as those TF genes whose target genes were over-represented among the PhaseCore genes. We identified 20 PhaseCoreTF genes (Table 1, Fig. 3C and Table S6), seven of them were also PhaseCore genes. The other 13 PhaseCoreTF genes were not PhaseCore genes, this may be caused by i) the TRN construction strategy that combined large number of expression profiles not used for PhaseCore gene identification, and ii) the fact that some TF genes’ activity could be regulated post-transcriptionally or post-translationally (Lelli et al., 2012). Compared with non-PhaseCoreTF genes, PhaseCoreTF genes have high-ranked PC1 values in the AC-PCA analysis (Mann-Whitney test, *P* = 1 × 10^−5^) and higher proportions of PRGs (binominal test, *P* = 2.7 × 10^−5^). These results indicated that PhaseCoreTF genes were rather closely associated with locust phase change than non-PhaseCoreTF genes.

**Table 1:** Enrichment of PhaseCoreTF genes in PhaseCore genes

GeneID	*P* value	Function description	Symbol	TF_Class	PhaseCore
LOCMI03018	2.03 × 10^−41^	LPS-induced tumor necrosis factor alpha factor	LITAF	zf-LITAF-like	Y
LOCMI17305	2.64 × 10^−22^	Hormone receptor 4	Hr4	zf-C4	N
LOCMI03017	3.02 × 10^−20^	LPS-induced tumor necrosis factor alpha factor	LITAF	zf-LITAF-like	Y
LOCMI03824	8.33 × 10^−15^	Zinc finger, BED-type predicted	ZBED	zf-BED	Y
LOCMI15104	2.23 × 10^−6^	Dwarfin sma-2	sma-2	MH2	N
LOCMI03971	2.43 × 10^−6^	PR domain zinc finger protein 1	Prdm1	zf-C2H2	Y
LOCMI04376	2.13 × 10^−5^	Myelin regulatory factor	Myrf	NDT80/PhoG	N
LOCMI16491	4.20 × 10^−5^	Probable nuclear hormone receptor HR3	Hr46	zf-C4	N
LOCMI17468	4.57 × 10^−5^	BTB/POZ domain-containing protein 19	BTBD19	BTB/POZ	N
LOCMI13163	1.24 × 10^−4^	Zinc finger and SCAN domain-containing protein 2	Zscan2	zf-C2H2	N
LOCMI16568	4.66 × 10^−4^	Zinc finger protein 729	ZNF729	zf-C2H2	N
LOCMI12694	8.55 × 10^−4^	Protein grainyhead	grh	CP2	N
LOCMI01314	8.55 × 10^−4^	ETS-related transcription factor Elf-1	Elf-1	Others	N
LOCMI15376	4.82 × 10^−3^	Zinc finger protein 768	ZNF768	zf-C2H2	Y
LOCMI07749	5.60 × 10^−3^	NFX1-type zinc finger-containing protein 1	Znfx1	zf-NF-X1	N
LOCMI07485	5.90 × 10^−3^	Protein ovo	ovo	zf-C2H2	Y
LOCMI09997	9.68 × 10^−3^	Zinc finger protein 808	ZNF808	BTB/POZ	N
LOCMI07877	1.45 × 10^−2^	Mediator of RNA polymerase II transcription subunit 8	MED8	MED	N
LOCMI06586	1.96 × 10^−2^	Protein max	MAX	bHLH	Y
LOCMI07477	2.10 × 10^−2^	Mesoderm posterior protein 1	MESP1	bHLH	N

To confirm the functions of PhaseCoreTF genes in the regulatory network, we performed gene ontology (GO) enrichment of TF target genes with PhaseCore genes as a background. We identified the enriched TFs for the terms enriched for PhaseCore genes (Fig. 2I). The functional classes involved in chitin metabolic process, aminoglycan metabolic process, carbohydrate metabolic process, and proteolysis, were mainly regulated by *LITAF* and *sma-2*. The TF *Myrf* regulated hemolymph JH binding; *grh* and *ZBED* regulated insect odorant-binding protein; and *Hr4* regulated insect cuticle protein (Fig. 3D).

We further investigated potential regulatory roles of PhaseCoreTF genes involved in the expression difference between two phases in locust brain tissue using Brain_Hou dataset (Fig. 3E). By target enrichment analysis, we found 12 PhaseCore TF genes regulating the DEGs (Table S7). According to the expression levels and known functions related to behaviour plasticity, we selected three TF genes for further functional verification, i.e., *Hr4*, *Hr46* and *grh*. The expression levels of these three TF genes were relatively higher than those of most other genes (Figs. 3F and S7).

### Functional verification of representative TFs

**Figure 4:**
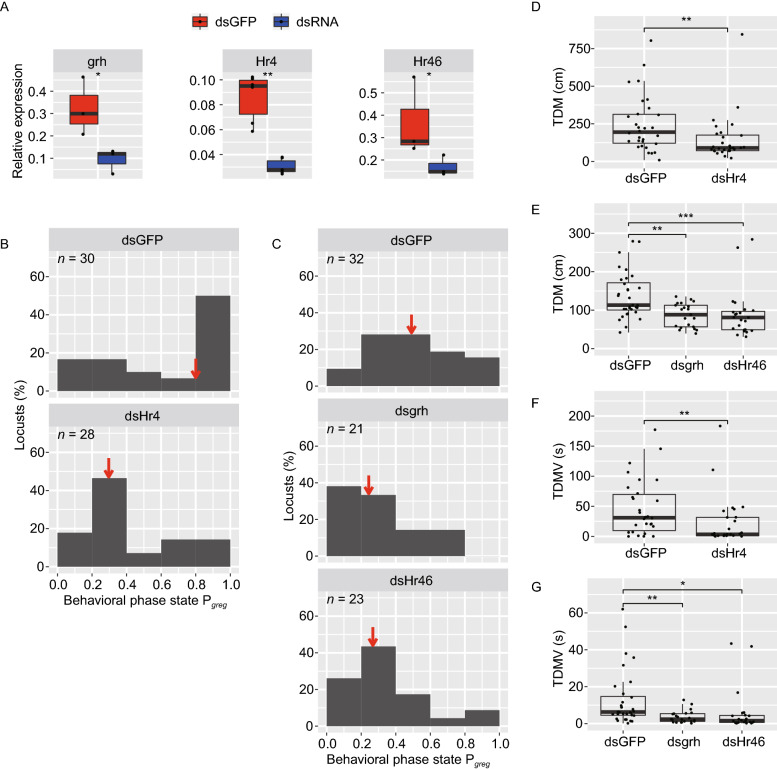
***Hr4***, ***Hr46*, and*grh*regulating locust phase behavior**. (A) Expression levels of three TF genes after RNA interference (RNAi). (B) and (C) Behavioral changes induced by RNAi of *Hr4* (B), *grh* and *Hr46* (C). The red arrows denote the median P_*greg*_ values. P_*greg*_ = 1 indicates full gregarious behavior, and P_*greg*_ = 0 indicates fully solitary behavior. (D) and (E) Total distance moved (TDM) 48h after injection of dsRNA of *Hr4*, *grh* and *Hr46*. (F and G) Total duration of movement (TDMV) 48 h after injection of dsRNA of *Hr4*, *grh* and *Hr46*. *P < 0.05, **P < 0.01 by Mann-Whitney test

Because hormone receptors have been reported to be critical for hormone regulating behavior plasticity (Pfaff and Joels, 2016), and *grh* was found to be involved in central nervous system development (Baumgardt et al., 2014), we carried out knock-down experiments to validate the functions of these three TFs (*Hr4*, *Hr46* and *grh*), followed by behavior tests and RNA-seq of brain tissues. When *Hr4* was knocked down by RNA interference (RNAi) in the fourth-instar gregarious nymphs (Fig. 4A), the P_*greg*_ value was significantly reduced towards solitary status (Mann-Whitney test, *P* = 0.024, Fig. 4B). Similar behavioral changes were observed for knockdowns of *Hr46* and *grh* (Mann-Whitney test, *P* < 0.005; Fig. 4A and 4B). Behavioral parameter analysis demonstrated that locust locomotor activity, including total duration of movement and total distance moved, were strongly suppressed by knockdowns of these three TFs separately (Fig. 4D–G). These results indicated that the three TFs could regulate phase change through influencing locust locomotor activity.

**Figure 5:**
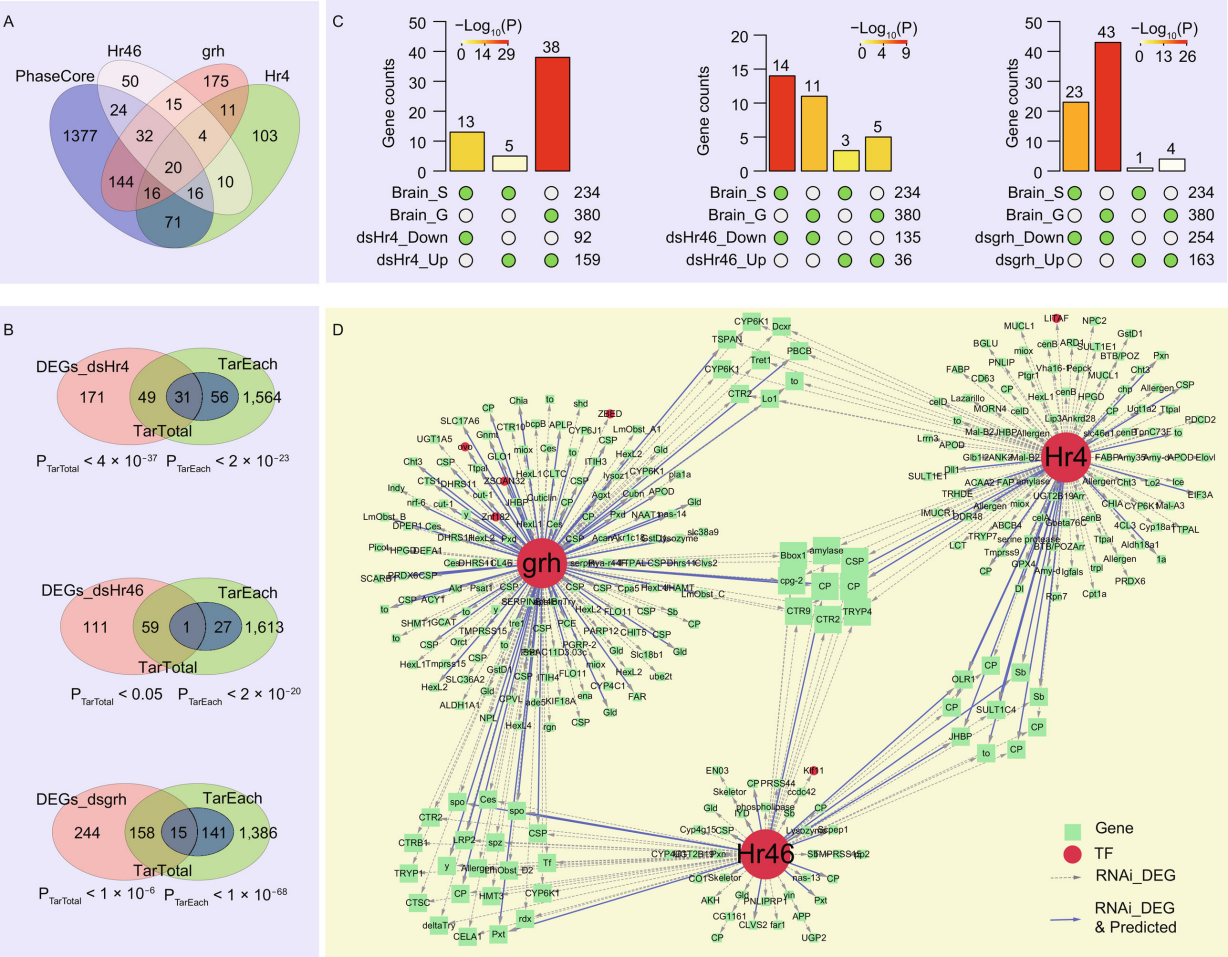
**RNA-seq revealed combinatorial regulations among*Hr4***, ***Hr46*and*grh***. (A) Venn diagram displaying the overlap among the three RNAi DEG lists and PhaseCore genes. (B) Venn diagram displaying the overlap among the DEGs from RNAi and target genes. Two target gene sets were used: the target genes through ensembling TF-Target pairs of total TF genes (TarTotal), and ensembling TF-target pairs of each TF gene (TarEach) (see MATERIALS AND METHODS). The hypergeometric test *P* value was calculated for these two target gene sets. (C) Bar chart that illustrates two sets intersections among four DEG lists in a matrix layout. The matrix of solid and empty circles at the bottom illustrates the “presence” (solid green) or “absence” (empty) of the gene sets in each intersection. The number to the right of the matrix indicates gene set size. The colored bars on the top of the matrix represent the intersection sizes, with the color intensity showing the *P* value significance. The DEGs in normal brain tissues were derived from Brain_Hou dataset. (D) Network of the DEGs from the RNAi of *Hr4*, *Hr46*, and *grh*. PhaseCore genes of the time course data with functional annotation are displayed. Red circles indicate TFs, and the green rectangles indicate no TFs. The edges with dashed lines indicate DEGs after RNAi, and the edges with solid lines indicate that the connections were supported by RNAi DEGs and target prediction

Transcriptome profiling of brain tissues in gregarious locusts treated by knockdowns of three TF genes, *Hr4*, *Hr46* and *grh*, showed that 251, 171, and 417 genes displayed differential expression levels compared with the GFP control (Fig. 5A and Table S8). Among these RNAi-induced DEGs, 124 were regulated by at least two TF genes. We found that a significant overlap between the predicted target genes of these three TF genes and RNAi-induced DEGs, which supported the accuracy of our target prediction methods (Fig. 5B). This result was confirmed with two target gene sets: one set had relatively strict criteria and relatively few genes (SuperExactTest, *P* < 1 × 10^−6^ except *Hr46*), and the other had relaxed criteria and more genes (SuperExactTest, *P* < 1 × 10^−20^ for all three TF genes). Of these RNAi-induced DEGs, 56, 33, and 71 genes were also differentially expressed in brain tissues between gregarious and solitary locusts (hypergeometric test, *P* < 1 × 10^−26^ for *Hr4* and *grh*, *P* < 1 × 10^−9^ for *Hr46*; Fig. 5C).

To illustrate possible regulatory coordination involving these three TF genes, we constructed a network that combined the RNAi-induced DEGs and predicted target genes (Fig. 5D). GO enrichment analysis revealed that these RNAi-induced DEGs were mainly associated with energy metabolisms, oxidation-reduction processes, and cellular structures (Table S9). Therefore, these results indicated that these three PhaseCoreTFs regulated locust phase change in a combinatorial manner.

### LocustMine database construction

**Figure 6:**
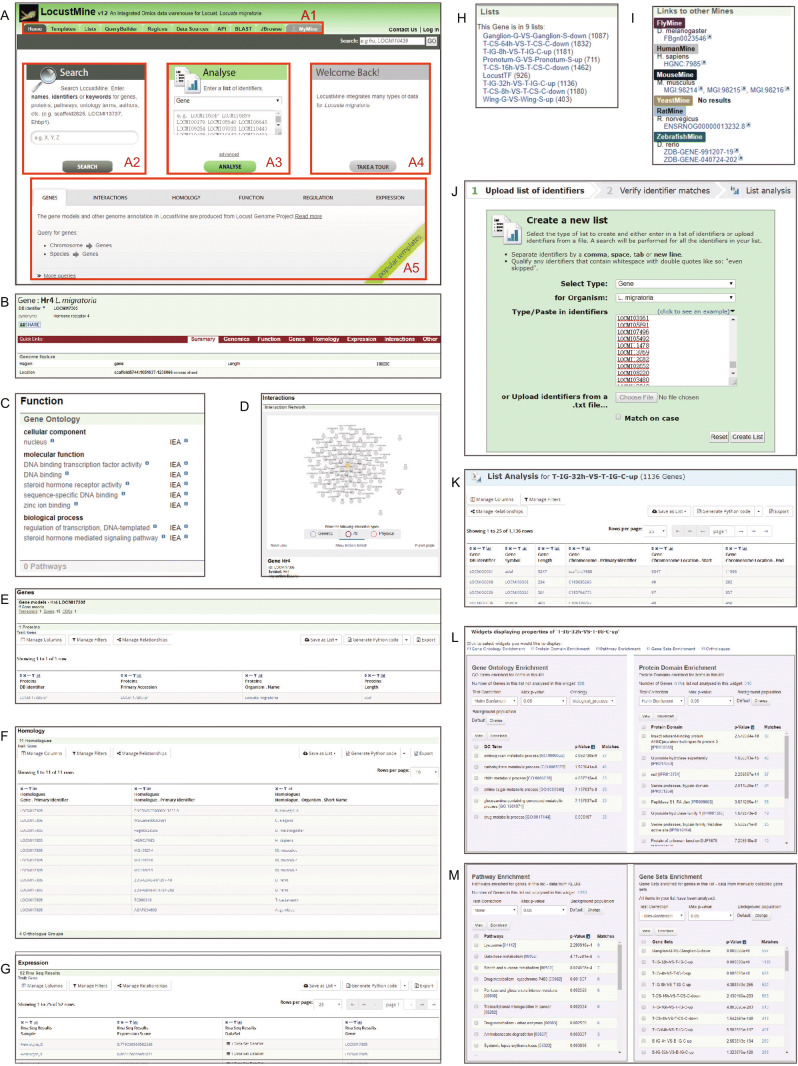
**LocustMine use case**. (A) LocustMine homepage. (A1) Quick visit to subsections, including BLAST and JBrowse. (A2) Enter a gene name to access the Gene page report. (A3) Enter a list of genes to perform GO and pathway enrichments. (A4) Take a tour will direct to a new page of LocustMine documentation. (A5) Popular template queries can be found here and under the Templates button at the top of the page. (B–I) Illustrate the report page of gene *Hr4* (http://locustmine.org:8080/locustmine/gene:LOCMI17305). (B) Header of gene report page, including quick link to several subsections. (C) Gene function, including gene ontology and pathways. (D) Interactions from PPI, co-expression and TF-Target. (E) Gene models and proteins. (F) Homology information. (G) Gene expression values from 52 samples. (H) Gene lists containing *Hr4*. (I) Links to the orthologues in other Mines. (J–M) Enrichment analysis for gene list T-IG-32h-VS-T-IG-C-up. The demo case could be accessed from the link: http://locustmine.org:8080/locustmine/bagDetails.do?scope=all&bagName=T-IG-32h-VS-T-IG-C-up. (J) Under *Lists* on the LocustMine homepage, users can manually enter or upload a list of genes for analysis. Here, we use the the public list T-IG-32h-VS-T-IG-C-up as example. (K) Screenshot of gene information of the list. (L) Gene ontology and protein domain enrichment. (M) Pathway enrichment and Gene Sets enrichment

To facilitate interpretation, searching, and visualization of the results of this study by other researchers, we constructed a database called LocustMine (http://www.locustmine.org:8080/locustmine). In addition to the previously published genome and CDS/protein sequences (Wang et al., 2014), LocustMine contains all gene expression data from the development, tissue, and time course datasets, the predicted TF-target data, and the co-expression network. LocustMine also links to the orthologues in six InterMine-based databases of model organisms, such as FlyMine (Lyne et al., 2007) and HumanMine (Smith et al., 2012). LocustMine is useful for performing gene set enrichment analysis, and currently supports GO, protein domain, and Kyoto encyclopedia of genes and genomes (KEGG) analysis. The homepage, report page of one gene and enrichment analysis of gene list were illustrated in Fig. 6.

## DISCUSSION

In this study, identification and analysis of PhaseCore and PhaseCoreTF genes demonstrated that there exist core transcriptional signatures of phenotypic plasticity in the migratory locust.

We re-analyzed all RNA-seq datasets by using the same pipeline and normalized the gene expression matrix that fed to AC-PCA using a quantile-normalization method (Pan and Zhang, 2018). Confounding factors has been removed through AC-PCA analysis. The LOO-CV was performed within datasets and CDV was performed inter datasets, which supports the robust of PhaseCore genes identified. The accuracy of PhaseCore gene was validated by two external RNA-seq datasets with three replications and some of them have been experimentally validated in previous studies. Moreover, these PhaseCore genes displayed distinct attributes similar to that of plasticity-related genes from other species. Therefore, we think, at least most, if not all, of the PhaseCore genes that we identified are reliable and confident. In spite of this, due to the complicated nature of locust phase change, we could only approximately predict the number of PhaseCore genes. More careful design of future experiment is needed to uncover the exact PhaseCore gene list.

PhaseCore genes can predict phase status of new transcriptome profiles from various spatiotemporal scales with higher accuracy. This finding implied that these genes can be used as molecular markers to identify alternative phase phenotypes (Sumner et al., 2018). PhaseCore genes displayed specific gene attributes, which have been reported in several other species, such as caste-biased genes in social insects (higher CpG o/e (Elango et al., 2009), lower DNA methylation level (Patalano et al., 2015), faster evolution rate (Hunt et al., 2011), lower percentage of genes with annotated function (Ferreira et al., 2013), lower co-expression network connectivity (Morandin et al., 2016), sex-biased genes in fruit flies and mice (higher tissue-specific expression level (Meisel, 2011)), and morph-biased genes in pea aphids (faster evolution rate (Purandare et al., 2014)). PhaseCore genes with faster evolution rates that rapidly expand genotypic space may be co-opted for new functions (Helantera and Uller, 2014). Consistently, PhaseCore genes included a large number more of genes with unknown functions, similar with the findings in wasps, in which 75% of the caste-biased genes were novel genes (Ferreira et al., 2013). Weak methylation levels could facilitate protein sequence variation (Simola et al., 2013) and contribute to expression flexibility via alternative transcription start sites, exon skipping, and transient methylation (Roberts and Gavery, 2012). Taken together, possessing these gene features, PhaseCore genes can rapidly expand their genotypic space and expression variation scope, which helps locust adapt to the changed environment.

PhaseCore genes covered several critical pathways mainly associated with signaling pathways, metabolic processes, and anti-oxidative processes. Many previous identified critical genes were top ranked in the PhaseCore gene list. For example, the signaling pathways comprised some genes, included *CSP*s, *takeout*s, and members of the JH pathway. *CSP* and *takeout* genes are involved in olfactory response to locust-emitted odours (Guo et al., 2011). The JH signaling pathway was suggested to regulate locust phase change by influencing body color, morphometric parameters, and reproduction (Kang et al., 2004; Tawfik, 2012). Moreover, two enzymes (*FAH* and *hgo*) are involved in the catecholamine metabolic pathway as a key regulator of phase change (Ma et al., 2011). Numerous metabolic process-related terms were enriched for PhaseCore genes, which is consistent with the fact that gregarious locusts exhibit more active and intensive energy consumption for long-distance marching or flight (Wang and Kang, 2014). Several anti-oxidative molecules, such as *APOD* (Dassati et al., 2014), *GPX4*, and *PRDX6* (Wang et al., 2014), can eliminate reactive oxygen species (ROS) produced by excessive energy metabolism (Apel and Hirt, 2004). Therefore, the present results combined with other studies support the convincible and reproducible findings of our previous studies by ESTs and RNA-seq (Chen et al., 2010; Wang et al., 2013; Wang et al., 2014). We provided a valuable list of 20 PhaseCoreTF genes responsible for locust phase change and predicted their possible functions (Fig. 3D). Some of these regulatory relationships were supported by previous studies. For example, *LITAF* overexpression was found to be associated with metabolic disorders in humans (Cardoso et al., 2018), whereas *sma-2* was demonstrated be involved in metabolic homeostasis (Shin et al., 2015). Additionally, many of these PhaseCoreTF genes have been reported to be involved in various phenotypic plasticity-related biological processes, such as development (*grh* (Baumgardt et al., 2014), *Hr4* (Mane-Padros et al., 2012) and *MESP1* (Liu, 2017)) and social behavior (*Hr46* (Wang et al., 2009)). In concert with PhaseCoreTF identification method, RNAi-induced knock-down of three TF genes, *Hr4, Hr46*, and *grh*, drove the behaviors of gregarious locusts into solitary phase behaviors, validating that the identified PhaseCoreTF genes are responsible for phase change of locusts. Therefore, the findings of PhaseCoreTF genes improve our understanding of the important roles of TF genes in regulating phenotypic plasticity.

We found a common set of genes among each DEG list after knockdown of three PhasecoreTF genes, *Hr4*, *grh*, and *Hr46*, suggesting that these PhaseCoreTFs might coordinate the same downstream signaling to regulate phase-related behaviors. The potential coordination of *Hr4* and *grh* had been reported during the process of abdominal pigmentation in *Drosophila melanogaster* (Rogers et al., 2014). In *Daphnia magna*, *Hr4* and *Hr46* potentially regulate *grh* by binding to its promoter region (Spanier et al., 2017). Several PhaseCore genes, such as *CSP*, *takeout*, *JHBP*, and *Bbox1*, were also included in the 124 genes whose expression levels were altered by the knockdowns of at least two TF genes. In particular, *Bbox1* was found to be regulated by all three TF genes. This gene catalyzes the formation of L-carnitine from gamma-butyrobetaine, which is the last step in the L-carnitine biosynthetic pathway (https://www.uniprot.org/uniprot/O75936). Carnitines were previously reported to be the key regulatory metabolites in locust behavioral transition (Wu et al., 2012). *OLR1*, regulated by both *Hr4* and *Hr46*, has been reported to reduce the released level of nitric oxide (Sawamura et al., 1997), which is a key gas neurotransmitter to activate phase-related locomotor activity (Hou et al., 2017). Therefore, these PRG changes are able to mediate the effects of three PhaseCoreTFs on phase-related behaviors of the locusts.

## MATERIALS AND METHODS

### Datasets

The RNA-seq data in this study were obtained from three migratory locust datasets (Figs. 1A, S1 and Table S1). The first dataset included data from six different developmental stages (egg, combined first and second instar, third instar, fourth instar, fifth instar, and adult) from both solitary and gregarious locusts (Chen et al., 2010) that can be found under accession number SRP002665 in the NCBI SRA database (https://www.ncbi.nlm.nih.gov/sra). The second dataset included data from various tissues and organs of both phases, including three tissues or organs from adult locusts (fat body, hemolymph, and antenna), and five tissues or organs from the fourth instar (antenna, brain, thoracic ganglia, wing, and pronotum). Among these tissues, data from the fat body (accession number SRP013742 in SRA) and pronotum (accession number PRJNA399053 in SRA) have been published (Wang et al., 2013; Yang et al., 2019). The data from brain and thoracic ganglia tissues were the zero time points from the time course datasets of these two tissues. The third dataset was from the time course experiments, which refers to CS and IG at six time points (0, 4, 8, 16, 32, and 64 h). The time course datasets for brain and thoracic ganglia tissue were released in this study (accession number PRJNA412119 in SRA). We also used a total of 129 samples, which included the above-mentioned samples and an additional 81 samples from various tissues (Samples information and expression values can be seen in Table S2), to construct the genome-wide TF-target network.

### RNA sequencing

Total RNA was extracted using TRIzol reagent (Invitrogen, Carlsbad, CA, USA) and treated with RNase-free DNase I. Poly(A) mRNA was isolated using oligo d(T) beads. First-strand complementary DNA was generated using random hexamer-primed reverse transcription, followed by synthesis of the second-strand cDNA using RNaseH and DNA polymerase I. Paired-end RNA-seq libraries were prepared following Illumina’s protocols and sequenced on the Illumina HiSeq 2000 platform in BGI-Shenzhen.

### RNA-seq data analysis

The quality distribution of the RNA-seq raw data were first checked using FastQC (v0.11.5, http://www.bioinformatics.babraham.ac.uk/projects/fastqc/); the low-quality and adaptor contaminated reads were filtered using Trimmomatic (v0.30; http://www.usadellab.org/cms/index.php?page=trimmomatic; parameters: “ILLUMINACLIP:/adaptor_sequence.fa:2:8:6 SLIDINGWINDOW:4:15 MINLEN:40”). The filtered reads were mapped to the *L*. *migratoria* reference genome (Wang et al., 2014) using TopHat2 (version 2.0.13) (Trapnell et al., 2009). HTSeq (v0.10.0, https://htseq.readthedocs.io) was used to calculate the read count. To reduce various biases, we further used the trimmed mean of M-values (TMM) method to eliminate the influence of differences in RNA output size between samples. Gene expression level was measured as reads per kilobase per million mapped reads (RPKM). To identify DEGs from the experiments with three replicates, R package edge was applied (Robinson et al., 2010). Adjustment for multiple testing-associated bias was performed by the Benjamini–Hochberg method. Expression ratio ≥ 2 and adjusted *P* value < 0.05 were used as the threshold for significance of gene expression differences.

### AC-PCA

AC-PCA was proposed for simultaneous dimension reduction and adjustment for confounding variation (Lin et al., 2016), and was demonstrated to be successfully applied under various conditions. In this study, the desired biological variation was the phase difference, whereas the confounding factors included the developmental stages, tissues, and time points after treatment. Data matrix X represented gene expression, with rows representing samples and columns representing genes. The raw RPKM values had 1 added to them and were then log_2_-transformed. Variation among the samples was quantile-normalized using the preprocessCore package (https://github.com/bmbolstad/preprocessCore). Quantile normalization has recently been validated a superior method for removing inter-study variation (Pan and Zhang, 2018). The columns were mean-centered. The confounding matrix Y was designed according to the description of the acPCA package user guide. In brief, for the development and tissue datasets, all of the samples from the same phase were considered biological replicates. For the time course datasets, the samples from the brain and thoracic ganglia tissues at the same time points in the same treatment process were considered biological replicates. Because the four datasets for the 64-h time points clustered together regardless of the treatments and tissues, we removed this time point prior to AC-PCA model construction. AC-PCA was performed using the acPCA package (v1.2) downloaded from https://github.com/linzx06/AC-PCA/tree/master/R_package. The *acPCA* function was performed with a linear kernel and the input Lambda parameter was tuned using the *acPCAtuneLambda* function.

We utilized the Borda algorithm with a median method to aggregate the three PC1 values from the three datasets to produce one summarized Borda gene list. This method first ordered the PC1 values from the three datasets separately, then calculated the median value of the ranks in the three datasets for each gene as Borda’s score. The genes were ranked according to their Borda’s score. The Borda algorithm was implemented in the R package TopKLists (https://cran.r-project.org/web/packages/TopKLists/index.html). To retain the directional information, we ran the Borda algorithm twice, once with gregarious phase-biased genes ranked at the top, and the other with solitary phase-biased genes ranked at the top. The two Borda lists were merged into one ranked final list by alternately selecting one gene from each of the two lists from top to bottom. The PhaseCore genes were defined as the top 1,700 genes in the Borda list.

To predict phase status based on the constructed AC-PCA model, we first preprocessed the raw expression data by subjecting them to log_2_ transformation, quantile normalization, and mean-centering. The loadings and the data matrix from AC-PCA and the new data matrix were fed to the predict function in the mixOmics package (https://CRAN.R-project.org/package=mixOmics). The tool *predict* then output the loadings of the new expression data. The loadings of the constructed model had a plus or minus sign, which represented the two phases. If the predicted loadings had the same sign as those in the constructed model and the phase status was also the same, we determined that the prediction was correct.

To perform LOO-CV and CDV, we split the top-ranked 15,000 genes from the four ranked gene lists (three dataset-specific gene lists and one Borda list) into 15, 30, and 150 bins with 1,000, 500, and 100 genes per bin and then performed validation for each bin. For LOO-CV, the prediction accuracy was calculated as the percentage of samples accurately predicted their phase status. We performed LOO-CV on three datasets based on the four gene lists separately. To perform CDV, each time, we select one dataset to train the AC-PCA model and predict phase status of the samples from other two datasets and calculate the prediction accuracy. We performed CDV on three datasets based on the four gene lists, separately.

### Gene features

#### Phase-related genes

We defined PRGs as those genes displayed differential expression between gregarious and solitary locusts in the above mentioned three datasets, development, tissues and phase transition time course, respectively (Fig. S5). These genes were identified using the method provided by Audic & Claverie (Audic and Claverie, 1997), which was developed for experiments without replicates.

#### Specific expression

The specific expression of genes was measured using the specific expression index τ (Liao and Zhang, 2006), which is defined as follows:

$$\uptau_{\text{i}} = \frac{{\sum\nolimits_{{{\text{j}} = 1}}^{\text{n}} {\frac{{1 - \log_{2} \left( {{\text{S}}_{{\left( {\text{i,j}} \right)}} + 1} \right)}}{{\log_{2} \left( {S_{{\left( {{\text{i}},{ \hbox{max} }} \right)}} + 1} \right)}}} }}{{{\text{n}} - 1}}$$

in which *n* is the number of samples surveyed, *S*(*i*,*j*) is the RPKM of gene *i* in sample *j*, and *S*(*i*,*max*) is the highest RPKM of gene *i* in n samples.

#### Network connectivity

The co-expression networks for development, tissue, brain time course, and ganglia time course datasets were separately constructed using genes with summarized raw RPKM > 1. The R package weighted gene co-expression network analysis (WGCNA) (https://labs.genetics.ucla.edu/horvath/CoexpressionNetwork/Rpackages/WGCNA/) was used to construct the co-expression networks. Detailed methodology is described in the TRN construction subsection. The network connectivity of one gene was defined as the sum of the topological overlap of this gene with all other genes in the network.

#### Evolution rate

The evolution rate was calculated by comparing the grasshopper *Oedaleus asiaticus*, which belongs to the same subfamily *Oedipodinae* as *L*. *migratoria*. Transcriptomic data of this species were downloaded from the NCBI SRA database (SRR IDs SRR2051024, SRR3372608, SRR3372609, and SRR3372610). The filtered clean reads were assembled using Trinity (v2.0.6, https://github.com/trinityrnaseq/trinityrnaseq/) with default parameters. To reduce redundancy, we further clustered the assembly into clusters and separately assembled each cluster using TGICL (v2.1, https://sourceforge.net/projects/tgicl/). To select one representative transcript for genes with multiple isoforms, all of the sequences from TGICL were reclustered using CD-HIT (v4.6.1, http://weizhongli-lab.org/cd-hit/), and the single longest representative sequence was selected for each cluster.

Reciprocal blast searching was performed using the protein sequences from *O. asiaticus* and *L*. *migratoria*. The reciprocal best hit pairs were used to calculate Ka/Ks. Protein sequence pair alignment was performed with muscle (v 3.8.31, https://www.drive5.com/muscle/) and then converted to CDS alignment using an in-house Perl script. Stop codons and nonsense codons were removed. KaKs_Calculator (v2.0, https://sourceforge.net/projects/kakscalculator2/) was used to calculate Ka/Ks.

#### *CpG o*/*e*

CpG o/e is defined as %CG / (%C × %G), where %CG = #CG / (L − 1) and L is the sequence length.

#### Methylation level

To calculate methylation level, reduced representation bisulfite sequencing data were used that were sequenced in the Locust Genome Project for gregarious and solitary brain samples (downloaded from SRA; accession number SRP031775). First, adaptor contamination and low-quality reads were filtered using trimmomatic (v0.22). The clean data were then mapped using bismark (v0.7.12; https://www.bioinformatics.babraham.ac.uk/projects/bismark/) with bowtie2 (http://bowtie-bio.sourceforge.net/bowtie2/index.shtml) for alignment. The aligned results were merged together for all samples, and the methylation level was calculated for every CG site using the R package methylKit (https://bioconductor.org/packages/methylKit). The average methylation level across all of the CG sites in the gene body was calculated as the methylation level at the gene level. To obtain more reliable results, genes with fewer than 19 CG sites were filtered. Finally, 9,168 genes with available methylation levels were selected.

#### DEGs from experiments with replicates

Two published datasets from fourth-instar gregarious and solitary locusts with three replicates were used here. The Brain_Hou dataset was from the brain tissue, in which gregarious individuals were injected with ddH_2_O and solitary individuals were injected with dsGFP (accession number SRP092214 in SRA) (Hou et al., 2017). The Pronotum_Yang dataset was from pronotum integument (accession number PRJNA399053 in SRA) (Yang et al., 2019). The RNA-seq data processing and DEGs detection were performed as above described.

### Functional enrichment analysis

Enrichment analyses of functional classes, including those of GO, InterPro domains, and KEGG pathways, for the supplied gene list were carried out based on an algorithm presented by GOstat (Beissbarth and Speed, 2004), with the whole annotated gene set being used as the background. The *P*-value of the enrichment score was determined using the chi-squared test. Fisher’s exact test was used when any expected value was below 5, which would have made the chi-squared test inaccurate. To adjust for multiple testing, we calculated the false discovery rate using the Benjamini-Hochberg method. The functional classes were removed if the enriched number of the genes was less than three.

### TF identification

To identify the TFs in the migratory locust, we used previously described methods (Weirauch and Hughes, 2011; Jin et al., 2014; Zhang et al., 2015) to search for locust proteins using the Pfam domain (Finn et al., 2014) and other protein family information using InterProScan (v5.2-45.0, ftp://ftp.ebi.ac.uk/pub/software/unix/iprscan/5/5.2-45.0/interproscan-5.2-45.0-64-bit.tar.gz). Each TF was classified into a particular TF family based on the interpro and Pfam ID, as previously described (Zhang et al., 2015). Several proteins without domain information were manually annotated.

### TRN construction

#### Overall TRN construction pipeline

It has demonstrated that integration of predictions from multiple TRN inference methods showed higher performance than any single inference method (Marbach et al., 2012). Our TRN construction strategy consulted those from the DREAM5 challenge. We selected eight widely used methods with high performance in the DREAM5 challenge (Greenfield et al., 2010; Marbach et al., 2012), available software, and representing the main TRN reconstruction algorithm categories, to construct the locust TRN. All of the genes with summarized raw RPKM > 1 across all 129 samples were used. The raw RPKM was preprocessed before network construction by log_2_ transformation after adding 1; then, the variation among the samples was quantile-normalized using the preprocessCore package (https://github.com/bmbolstad/preprocessCore). Because these transcriptome datasets (development, tissue, and time course datasets for brain and thoracic ganglia) were produced from various tissues and development stages, their heterogeneity caused problems with using all of the data for some tools. Therefore, we split the data to run the tools separately when necessary. The eight methods were ARACNE, CLR (http://bioconductor.org/packages/minet), GENIE3 (http://bioconductor.org/packages/GENIE3/), LeMoNe (http://bioinformatics.psb.ugent.be/beg/tools/lemone), WGCNA, Inferelator (https://sites.google.com/a/nyu.edu/inferelator/home), TIGRESS (http://projets.cbio.mines-paristech.fr/~ahaury/svn/dream5/html/index.html), and GGM (https://cran.r-project.org/web/packages/GeneNet/index.html). For each method, TF-target gene pairs were arranged in decreasing order according to their regulatory strength. The top 100,000 TF-target pairs from the eight methods were aggregated using the Borda algorithm, which was implemented in the R package TopKLists (https://cran.r-project.org/web/packages/TopKLists/index.html). The top 100,000 TF-target pairs of the aggregated pair list were used to construct the TRN. However, when we checked the target degree distribution, we found that 97 target genes were regulated by all of the 876 TF genes in the network. Therefore, we manually filtered these target genes, including three TF genes, and reconstructed the network.

#### Eight TRN construction methods

The mutual information-based algorithms ARACNE and CLR were implemented in the R package minet (http://bioconductor.org/packages/minet) and run with the default parameters. All 129 samples were used. The scores that measured the regulation strength between the TF and all genes were used to rank the TF-target pairs.

The tree-based method GENIE3 was also run using all 129 samples with the default parameters. The score assigned by this algorithm was used to rank the TF-target pairs.

To run the module-based LeMoNe method, we first defined gene clusters that showed similar expression trends across samples. The preprocessed RPKM values, subjected to log_2_ transformation and quantile normalization, were clustered using the *K*-means algorithm implemented in the R package stats command *kmeans* with the default parameters. The number of clusters (*K*) was chosen to minimize the Bayesian information criterion (BIC) (Hastie et al., 2009). The BIC is a function of *K* represented as *BIC*(*K*),

$$BIC\left( K \right) = \sum\limits_{{l = 1}}^{N} {\sum\limits_{{j = 1}}^{M} {\left( {\frac{{x_{{lj}} - c_{{k_{{l,j}} }} }}{{\sigma _{\varepsilon } }}} \right)} } + \log \left( N \right) \times M \times K$$

where *k*_*l*_ was the cluster to which the *l*^*th*^ gene was assigned, and $c_{{k_{l,j} }}$ was the *j*^*th*^ coordinate of the centroid of the *k*^*th*^ cluster in the space of expression measurements. *N* was the number of DEGs in each dataset, and *M* was the number of samples for which clustering was performed. $\sigma_{\varepsilon }^{2}$ is the mean intra-cluster variance evaluated at *K* = 3. The *K*-means clustering was carried out for integer values 3 ≤ *K* ≤ 100; for 1,000 iterations at each value of *K*, the optimal clustering of *K* was determined based on the lowest BIC value.

Clusters were produced for each of the four datasets. A score was assigned to every cluster for each TF gene by the LeMoNe algorithm. We used this score to represent the regulation strength of gene members of that cluster for this TF. The TF-target pairs from the four datasets were aggregated using the Borda algorithm, and the rank given by the Borda algorithm was used as the output from the LeMoNe method.

The correlation-based WGCNA method was run for each of the four datasets. The signed adjacency matrix was calculated with power 14, 12, 5, and 8 for development, tissue, and brain and ganglia time course datasets. For each pair of genes, their topological overlap was calculated based on the adjacency matrix and used to measure the correlation between them. To summarize the TF-target correlation, the ranked TF-target correlations from each dataset were merged using the Borda algorithm.

The *t*-test-based method Inferelator was downloaded from https://github.com/ChristophH/Inferelator and run on the R platform with the default parameters using all 129 samples. The score was used to order the TF-target pairs.

The regression-based method TIGRESS (v2.1) was run in MATLAB (v2007) using all 129 samples. The algorithm was run with the parameters *R* = 1,000, alpha = 0.3, and L = 5 at the stability selection step, and the area method was used to score the edges. The score was used to rank the TF-target pairs.

The graphical Gaussian-based GGM algorithm was implemented in the R package GeneNet and run using all 129 samples. First, partial correlation estimation was performed with a dynamic method. Second, the significance was tested based on regulatory direction. The TF-target pairs were ordered according to their significant Q values.

#### TRN for each TF

The above mentioned TRN was constructed based on the top TF-target connections by aggregating all TF genes. However, for some TF genes, the number of their target genes could be very small because of their lower-ranking TF-target connections. To construct a comparable TRN for each TF gene, we used the same ensemble method for each TF gene and selected the top 1,700 TF-target connections to construct the TRN.

### PhaseCoreTF gene analysis

Significance of enrichment of the PhaseCore gene targets was tested using a hypergeometric distribution. TF genes with at least three enriched target genes were retained. Adjustment for multiple testing was performed using the Benjamini-Hochberg method. To test the specific function of the PhaseCoreTF genes, we performed GO enrichment analysis with the PhaseCore genes as a background.

### qPCR

qPCR for *Hr4*, *Hr46*, and *grh* was performed using a SYBR Green kit on a LightCycler 480 instrument (Roche). *RP49* was used as internal reference. The PCR primer sequences are shown in Table S10. The 2^−ΔΔCt^ method was used to determine relative mRNA abundance for the surveyed samples.

### RNAi and behavioral tests

The dsRNA sequences for three TF genes *Hr4*, *Hr46* and *grh* were prepared using the T7 RiboMAX Express RNAi system (Promega). dsRNA was microinjected into the brains of fourth-instar locusts (1 μg/locust). dsGFP-RNA was used as the control. The behaviors of test locusts were measured 48 h after injection. The behavioral test was performed in a rectangular arena (40 cm × 30 cm × 10 cm) that contained three chambers, as previously described (Guo et al., 2011; Hou et al., 2017). Two smaller chambers (7.5 cm × 30 cm × 10 cm) were at either end; one contained 30 fourth-instar gregarious locusts as a stimulus group, and the other was left empty. Locust behaviors were recorded for 300 s by an EthoVision video tracking system and analyzed according to the binary logistic regression model. P_*greg*_ was calculated as eη / (1 + eη); η = −2.11 + 0.005  × attraction index (AI) + 0.012 × total distance moved + 0.015 × total duration of movement; AI = total duration in stimulus area – total duration in area opposite the stimulus; this parameter represents the extent to which the tested animals are attracted by the stimulus group. After behavioral tests, the brains of these locusts were collected. Three independent replicates were performed for each treatment.

### Statistics and visualization

All statistical analyses were performed using *R* (https://www.r-project.org/). Venn diagrams were plotted using the R package VennDiagram (https://cran.r-project.org/web/packages/VennDiagram/index.html). The network was presented using Cytoscape (v3.6.0, https://cytoscape.org/). Most of the graphs were produced using the R package ggplot2 (https://cran.r-project.org/web/packages/ggplot2/index.html). The overlap between RNAi DEGs and DEGs from gregarious and solitary locust brain tissues was tested and visualized using SuperExactTest package in R (https://github.com/mw201608/SuperExactTest).

### Data availability

The raw reads generated and/or analyzed during the current study are available in the NCBI/SRA repository under accession IDs SRP119014 for IG and CS time course, and SRP167424 for RNAi of the three TF genes. The gene expression scores in RPKM for the samples from development, tissues and phase transition time courses are provided in Table S2, and the gene expression scores for the samples from the RNAi of three TF genes are provided in Table S8. The gene expression scores are also available on LocustMine (http://www.locustmine.org:8080/locustmine).

## Electronic supplementary material

Below is the link to the electronic supplementary material.

Supplementary material 1 (XLSX 29543 kb)

Supplementary material 2 (PDF 1522 kb)
