# Correction to: Core transcriptional signatures of phase change in the migratory locust

**DOI:** 10.1007/s13238-019-00688-4

**Published:** 2020-02-06

**Authors:** Pengcheng Yang, Li Hou, Xianhui Wang, Le Kang

**Affiliations:** 1 Beijing Institutes of Life Science, Chinese Academy of Sciences, Beijing, 100101 China; 2 State Key Laboratory of Integrated Management of Pest Insects and Rodents, Institute of Zoology, Chinese Academy of Sciences, Beijing, 100101 China

## Correction to: Protein & Cell (2019) 10:883 10.1007/s13238-019-0648-6

In the original publication the photo of the gregarious adult locust in Fig. 1A is incorrect. The correct photo of adult migratory locust is provided in this correction.

**Figure 1:**
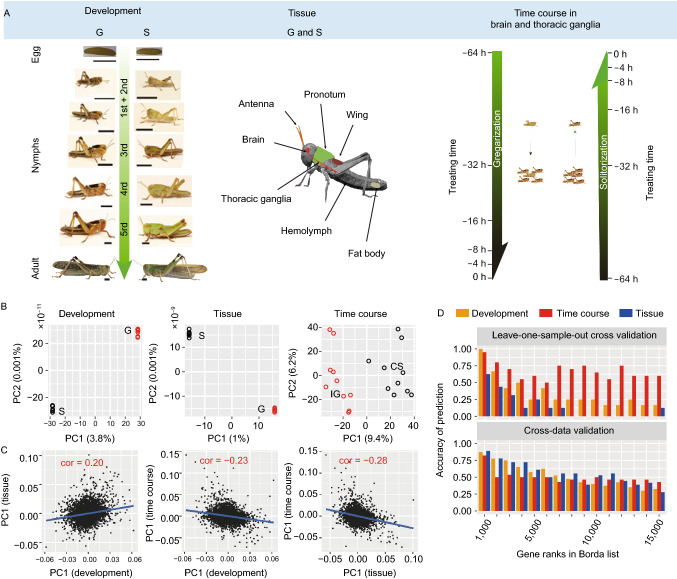
**PhaseCore gene identification**. (A) Experimental design of this study. Left: developmental stages from eggs to adults. Scale bars = 5 mm. Middle: various tissues, including three tissues from adult locust (fat body, hemolymph, and antenna), and five tissues from the fourth instar nymphs (antenna, brain, thoracic ganglia, wing, and pronotum). Right: the time courses of phase change (i.e., gregarization and solitarization) with two brain and thoracic ganglia tissues at six time points (0, 4, 8, 16, 32, and 64 h). (B) Samples from gregarious (G) and solitary locusts (S), and CS and IG locusts classified using the AC-PCA method for developmental, tissue, and time course datasets. One circle represents one sample. Blue represents typical or crowded solitary locusts, and red represents typical or isolated gregarious locusts. (C) Scatterplots and Pearson’s correlation (marked in red) of pairs of the PC1 values from the three datasets. Lines were fitted using least-squares linear regression. (D) Accuracy distribution of leave-one-out cross validation (LOO-CV) and cross-dataset validation (CDV) for the three datasets using Borda gene list. Only the top 15,000 genes were considered. These genes were divided into 15 bins with 1,000 genes in each bin. The accuracy was calculated for each bin

