# Supplementary material for: Core transcriptional signatures of phase change in the migratory locust
Source: Protein Cell. 2019 Jul 10;10(12):883–901. doi: 10.1007/s13238-019-0648-6 (PMC6881432; doi:10.1007/s13238-019-0648-6)
Supplement: Supplementary file 2 — Supplementary material 2 (PDF 1522 kb) [file 13238_2019_648_MOESM2_ESM.pdf]

# Supplementary Figures

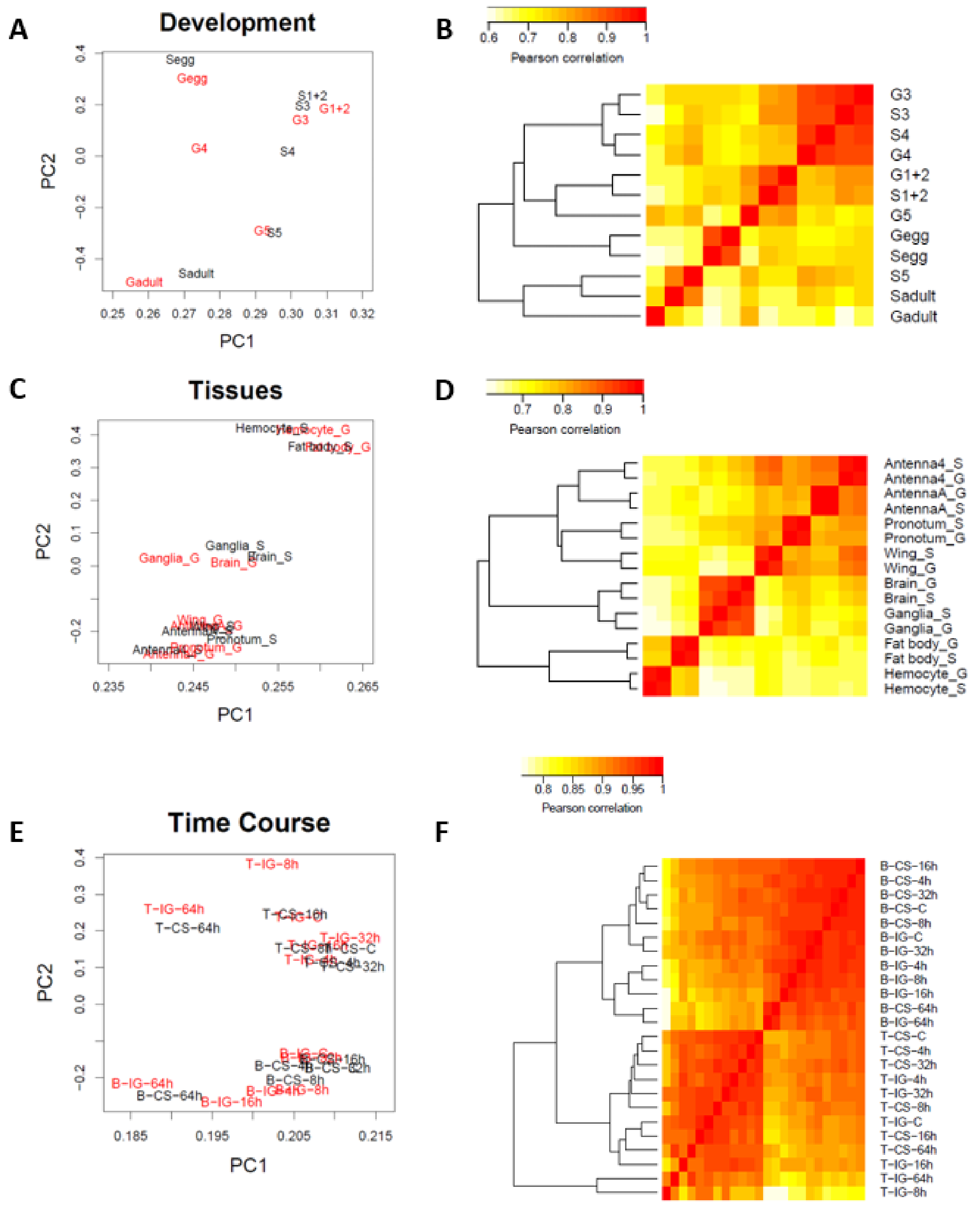


**Supplementary Figure S1. Expression clustering of three transcriptome datasets.** Principal component analysis (A, C, E) and hierarchical clustering (B, D, F) for development (A, B), tissue (C, D), and time course (E, F) datasets. G, gregarious locusts; S, solitary locusts; B, brain; T, thoracic ganglia; CS, crowding of solitary locusts; IG, isolation of gregarious locusts.

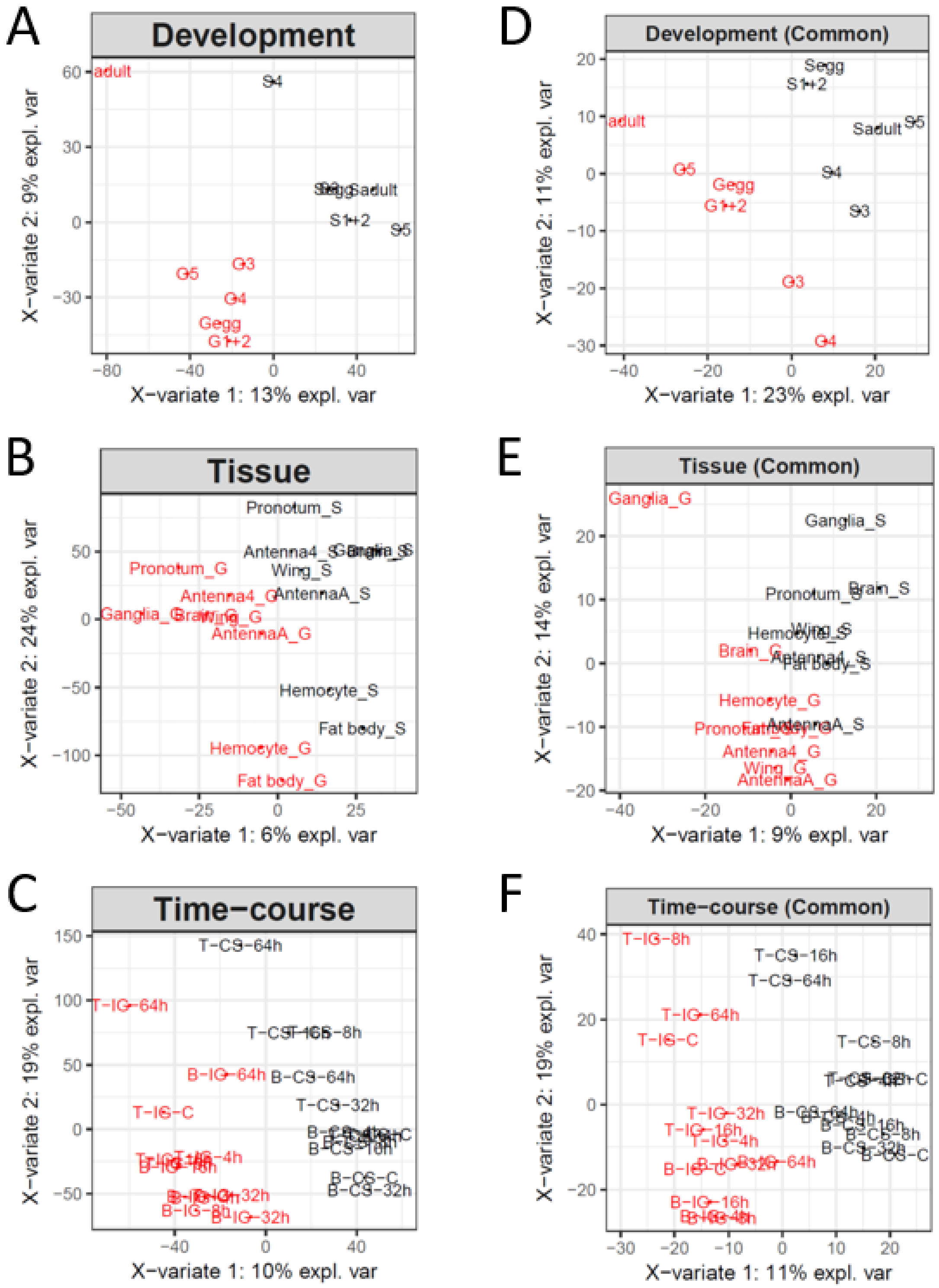


**Supplementary Figure S2. Supervised partial least square regression classification of locust transcriptome profiles from two phases.** (A–C) All genes. (D–F) Common genes among three datasets. (A, D) Development, (B, E) tissue, and (C, F) time course datasets.

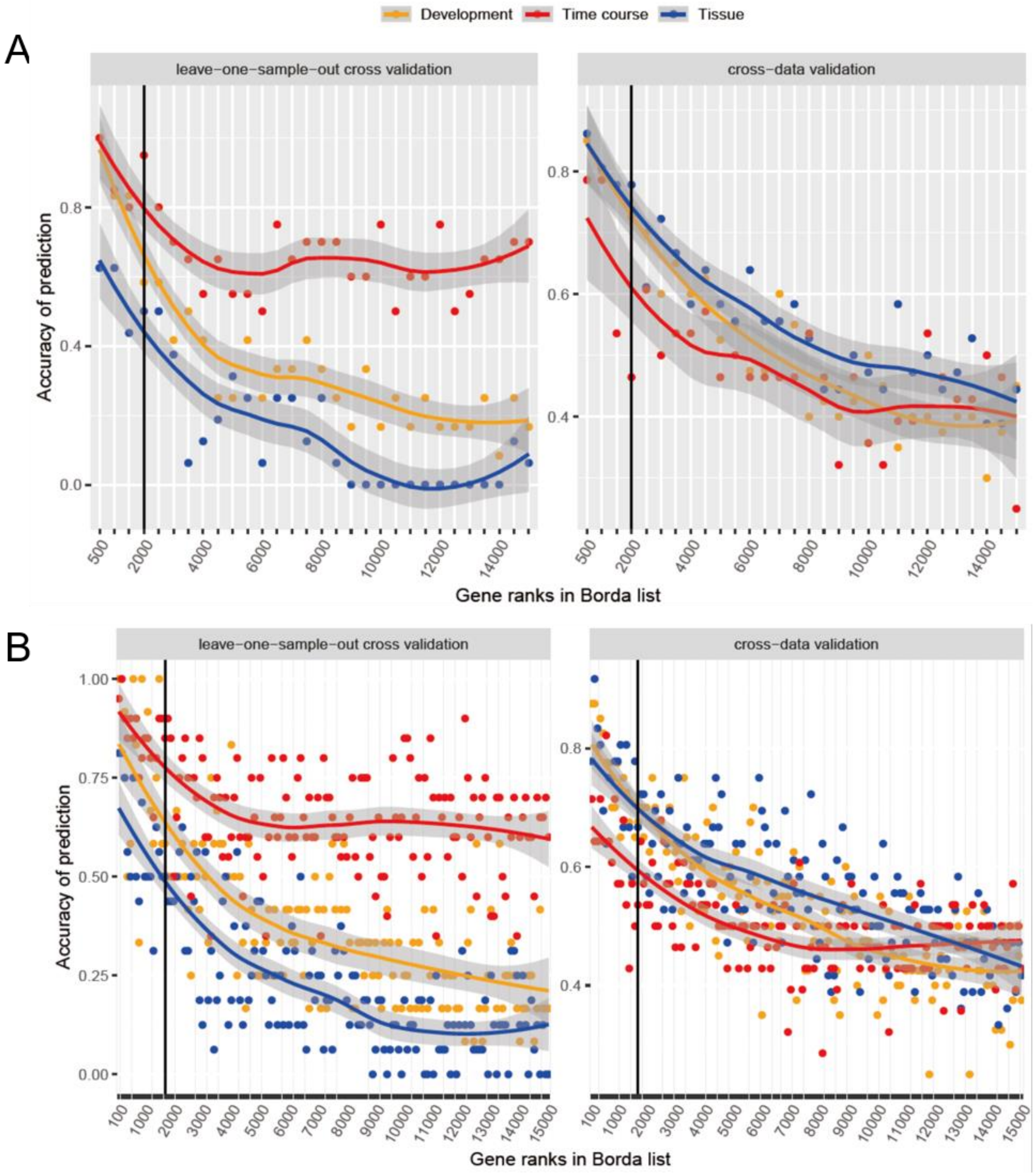


**Supplementary Figure S3 Accuracy distribution of leave-one-sample-out cross validation (LOO-CV) and cross-dataset validation (CDV) for the three datasets using Boda gene list.** The gene list was split into (A) 30 bins with 500 genes in each bin or (B) 150 bins with 100 genes in each bin. The black vertical lines denote the 17$^{th}$ bin in (A) and 4$^{th}$ bin in (B).

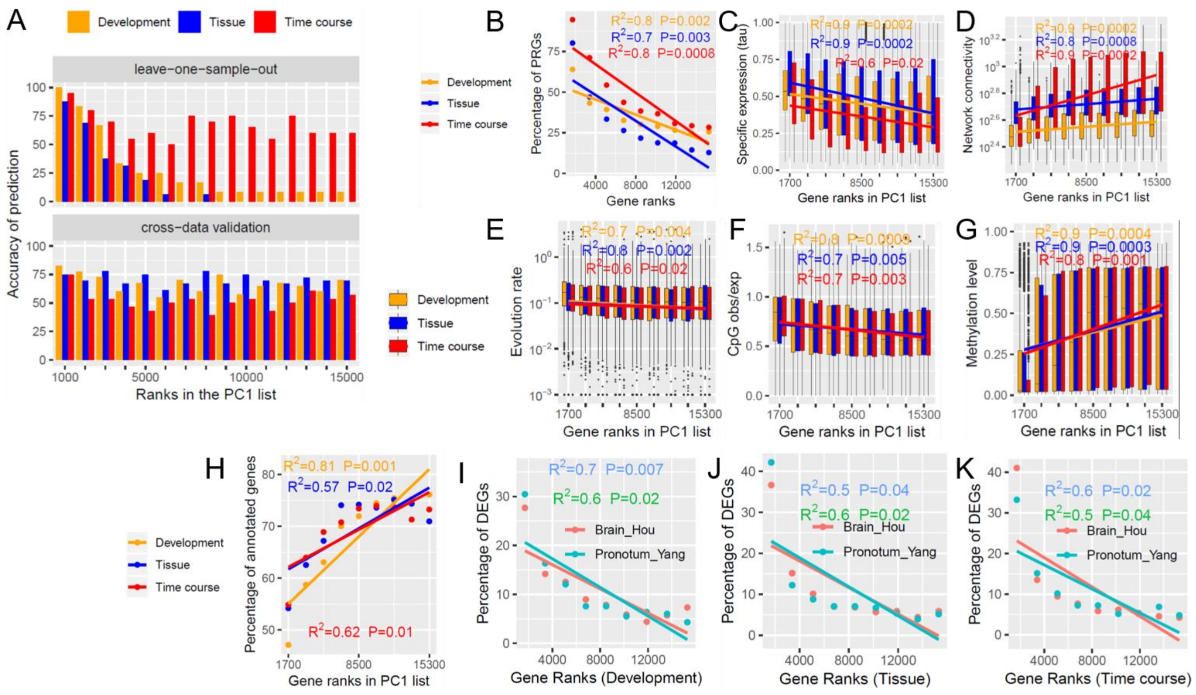


**Supplementary Figure S4 Gene attributes of the genes binned according to PC1 values of three datasets.** (A) Accuracy distribution of leave-one-out cross validation (LOO-CV) and cross-dataset validation (CDV). Only the top 15000 genes were considered. These genes were divided into 15 bins with 1000 genes in each bin. The genes were ranked according to absolute PC1 values from three datasets. The genes ranked higher in the list displayed (B) higher percentage of PRGs, (C) higher specific expression level, (D) lower network connectivity in the co-expression network, (E) faster evolution rate, (F) higher CpG o/e, (G) lower methylation level, (H) lower percentage of genes with known function and higher percentage of DEGs from two experiments with three replicates in (I) development, (J) tissue and (K) time course datasets, respectively.

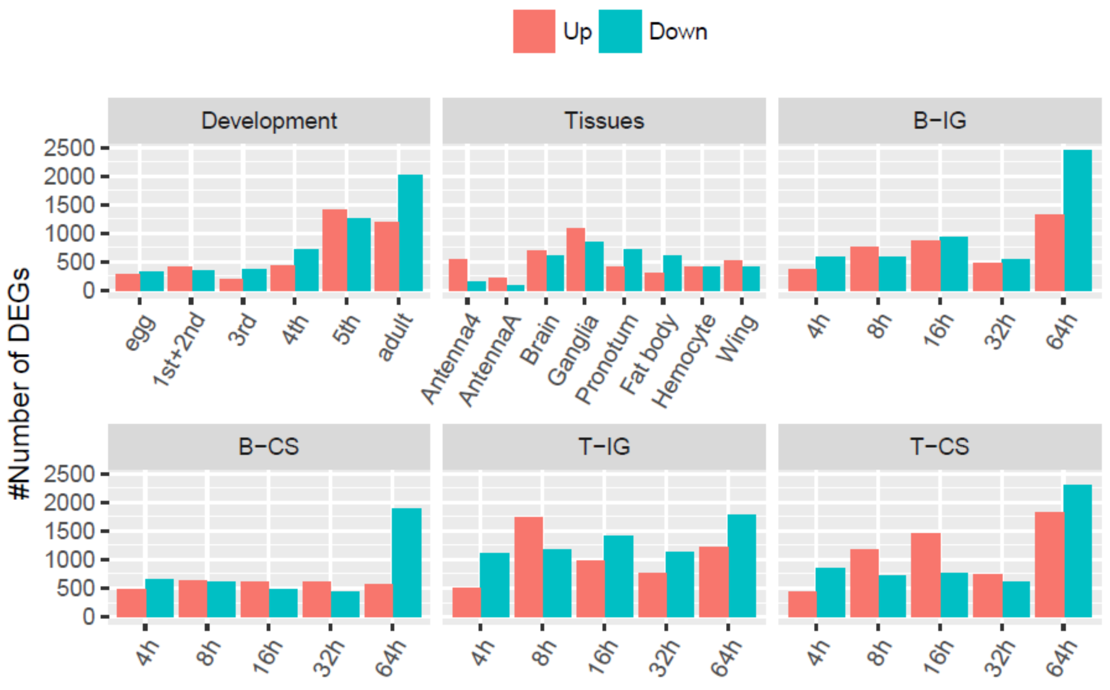


**Supplementary Figure S5. Phase-related genes in development, tissue, phase transition time course datasets.** Red denotes the number of highly expressed genes in gregarious locusts compared with solitary locusts in the development and tissue datasets, or in time points of treatments compared with the zero point in time course datasets. Antenna4: antenna tissue from the fourth instar; AntennaA: antenna tissue from adult; B: brain; T: thoracic ganglia; CS: crowding of solitary locusts; IG: isolation of gregarious locusts.

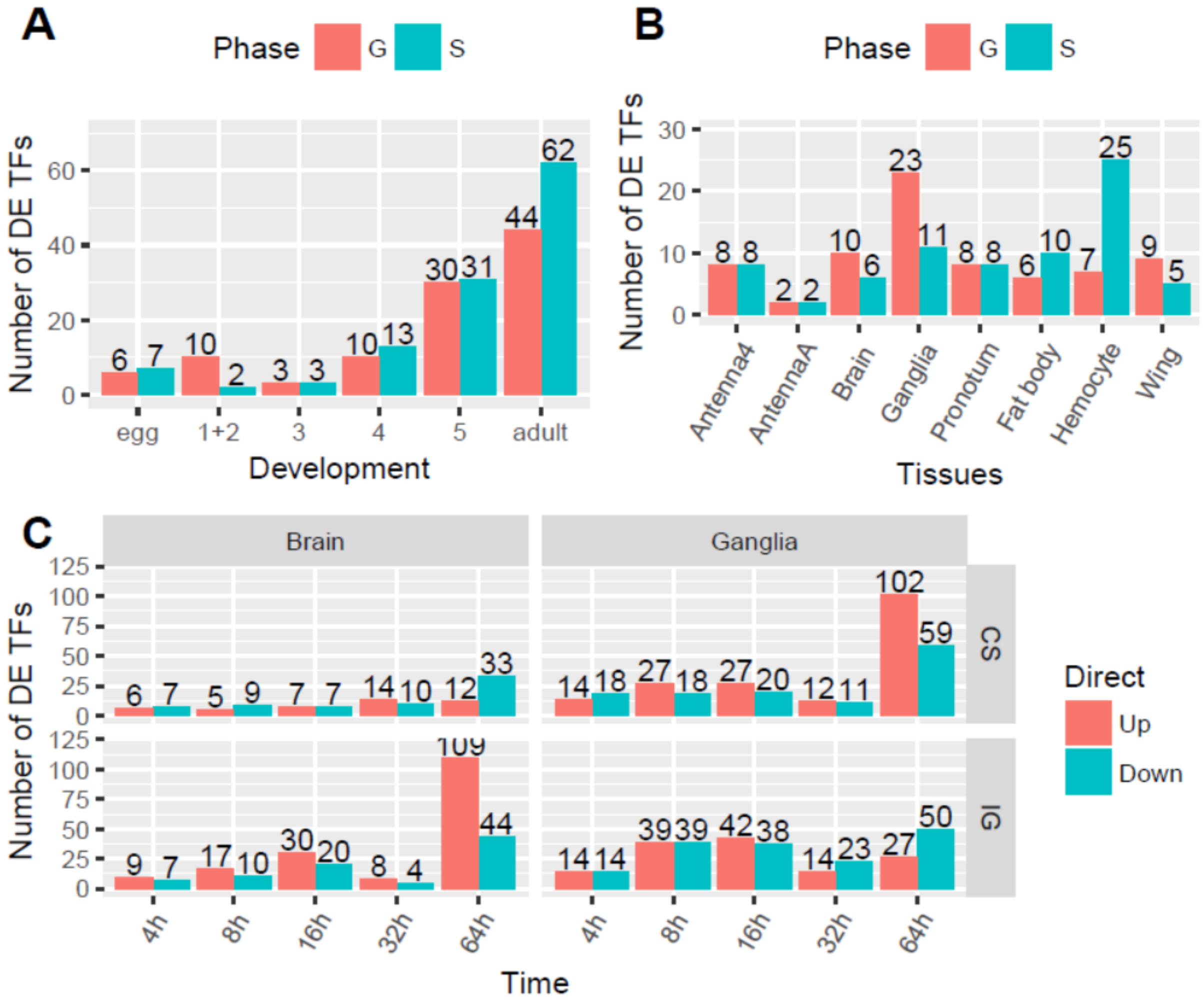


**Supplementary Figure S6. Numbers of phase-related genes among TF genes across the (A) development, (B) tissue, and (C) time course datasets.** Red represents the gregarious samples and upregulated genes in time course datasets, and blue represents the solitary samples and downregulated genes in time course datasets.

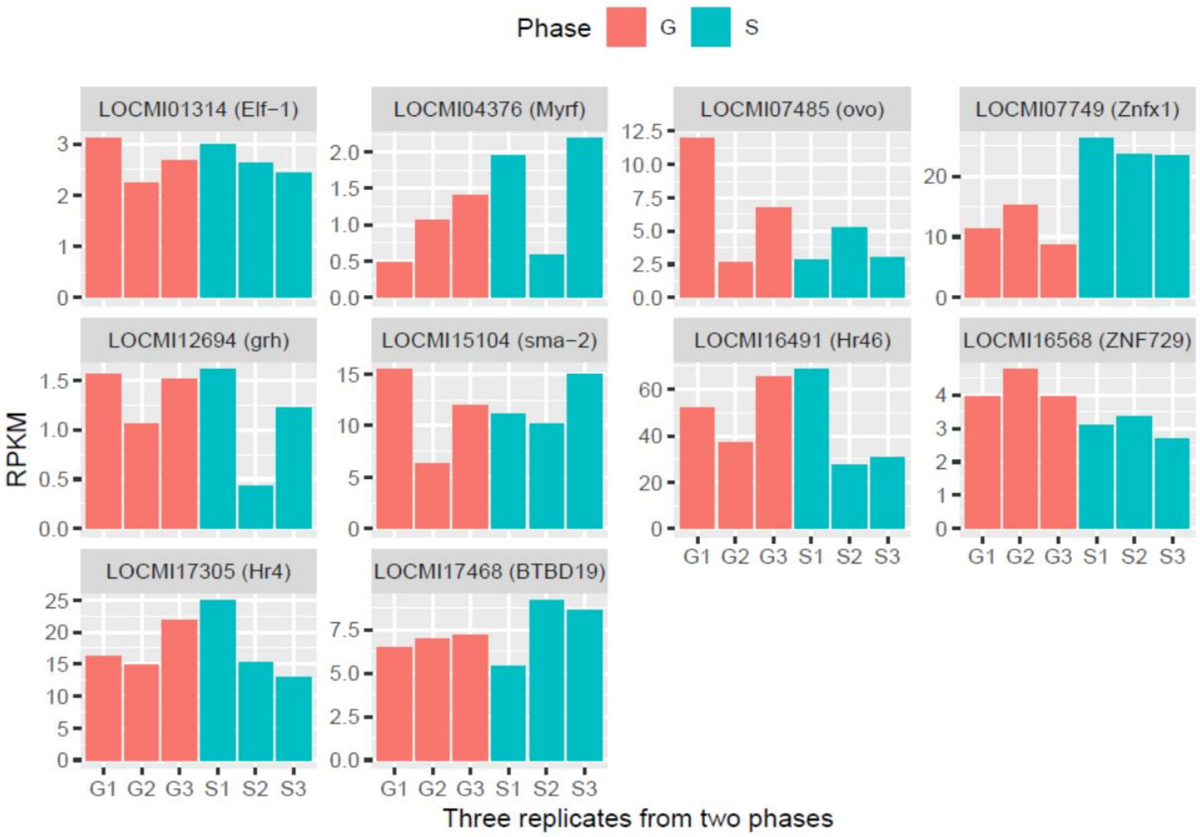


**Supplementary Figure S7. Expression levels of PhaseCoreTF genes enriched in the DEGs from Brain_Hou dataset.**

# Supplementary Table list:

**Supplementary Table S1.** Three datasets used in this study.

**Supplementary Table S2.** Gene expression scores in RPKM and tissue information for the samples from development, tissues, phase transition time courses and additional 81 samples.

**Supplementary Table S3.** List of PhaseCore genes and their functional annotation.

**Supplementary Table S4.** Functional class enrichment of the PhaseCore genes.

**Supplementary Table S5**. PhaseCore genes involved in JH signaling pathway.

**Supplementary Table S6**. PhaseCoreTF-PhaseCore gene pairs.

**Supplementary Table S7**. Enriched PhaseCoreTF genes in the DEGs between two phases in Brain_Hou dataset.

**Supplementary Table S8**. Gene expression scores in RPKM for the samples from the RNAi of three TF genes, *Hr4*, *Hr46* and *grh*, respectively.

**Supplementary Table S9**. Gene ontology enrichment of the differentially expressed genes from three RNAi experiments.

**Supplementary Table S10.** Primer sequences used for qPCR and RNAi experiments.
